# Supplementary material for: OpenMM 7: Rapid development of high performance algorithms for molecular dynamics
Source: PLoS Comput Biol. 2017 Jul 26;13(7):e1005659. doi: 10.1371/journal.pcbi.1005659 (PMC5549999; doi:10.1371/journal.pcbi.1005659)
Supplement: S1 Source Code — (ZIP) [file pcbi.1005659.s002.zip › OpenMM-7.0.1-Source/docs/api-python/generated/simtk.openmm.openmm.DrudeForce.html]

DrudeForce — OpenMM 7.0.1.dev-5e86c4f documentation


# DrudeForce¶

*class* `simtk.openmm.openmm.``DrudeForce`(*\*args*)¶
:   This class implements forces that are specific to Drude oscillators. There are two distinct forces it applies: an anisotropic harmonic force connecting each Drude particle to its parent particle; and a screened Coulomb interaction between specific pairs of dipoles. The latter is typically used between closely bonded particles whose Coulomb interaction would otherwise be fully excluded.

    To use this class, create a DrudeForce object, then call addParticle() once for each Drude particle in the System to define its parameters. After a particle has been added, you can modify its force field parameters by calling setParticleParameters(). This will have no effect on Contexts that already exist unless you call updateParametersInContext(). Likewise, call addScreenedPair() for each pair of dipoles (each dipole consisting of a Drude particle and its parent) that should be computed.

    `__init__`(*self*) → DrudeForce¶
    :   \_\_init\_\_(self, other) -> DrudeForce

        Create a DrudeForce.

    Methods

    |  |  |
    | --- | --- |
    | `__init__`((self) -> DrudeForce) | \_\_init\_\_(self, other) -> DrudeForce |
    | `addParticle`((self, particle, particle1, ...) | Add a Drude particle to which forces should be applied. |
    | `addScreenedPair`((self, particle1, particle2, ...) | Add an interaction to the list of screened pairs. |
    | `getForceGroup`((self) -> int) | Get the force group this Force belongs to. |
    | `getNumParticles`((self) -> int) | Get the number of particles for which force field parameters have been defined. |
    | `getNumScreenedPairs`((self) -> int) | Get the number of special interactions that should be calculated differently from other interactions. |
    | `getParticleParameters`(self, index) | Get the parameters for a Drude particle. |
    | `getScreenedPairParameters`(self, index) | Get the force field parameters for screened pair. |
    | `setForceGroup`(self, group) | Set the force group this Force belongs to. |
    | `setParticleParameters`(self, index, particle, ...) | Set the parameters for a Drude particle. |
    | `setScreenedPairParameters`(self, index, ...) | Set the force field parameters for screened pair. |
    | `updateParametersInContext`(self, context) | Update the particle and screened pair parameters in a Context to match those stored in this Force object. |
    | `usesPeriodicBoundaryConditions`((self) -> bool) | Returns whether or not this force makes use of periodic boundary conditions. |

    `getNumParticles`(*self*) → int¶
    :   Get the number of particles for which force field parameters have been defined.

    `getNumScreenedPairs`(*self*) → int¶
    :   Get the number of special interactions that should be calculated differently from other interactions.

    `addParticle`(*self*, *particle*, *particle1*, *particle2*, *particle3*, *particle4*, *charge*, *polarizability*, *aniso12*, *aniso34*) → int¶
    :   Add a Drude particle to which forces should be applied.

        |  |  |
        | --- | --- |
        | Parameters: | - **particle** (*int*) – the index within the System of the Drude particle - **particle1** (*int*) – the index within the System of the particle to which the Drude particle is attached - **particle2** (*int*) – the index within the System of the second particle used for defining anisotropic polarizability. This may be set to -1, in which case aniso12 will be ignored. - **particle3** (*int*) – the index within the System of the third particle used for defining anisotropic polarizability. This may be set to -1, in which case aniso34 will be ignored. - **particle4** (*int*) – the index within the System of the fourth particle used for defining anisotropic polarizability. This may be set to -1, in which case aniso34 will be ignored. - **charge** (*double*) – The charge on the Drude particle - **polarizability** (*double*) – The isotropic polarizability - **aniso12** (*double*) – The scale factor for the polarizability along the direction defined by particle1 and particle2 - **aniso34** (*double*) – The scale factor for the polarizability along the direction defined by particle3 and particle4 |
        | Returns: | the index of the particle that was added |
        | Return type: | int |

    `getParticleParameters`(*self*, *index*)¶
    :   Get the parameters for a Drude particle.

        |  |  |
        | --- | --- |
        | Parameters: | **index** (*int*) – the index of the Drude particle for which to get parameters |
        | Returns: | - **particle** (*int*) –   the index within the System of the Drude particle - **particle1** (*int*) –   the index within the System of the particle to which the Drude particle is attached - **particle2** (*int*) –   the index within the System of the second particle used for defining anisotropic polarizability. This may be set to -1, in which case aniso12 will be ignored. - **particle3** (*int*) –   the index within the System of the third particle used for defining anisotropic polarizability. This may be set to -1, in which case aniso34 will be ignored. - **particle4** (*int*) –   the index within the System of the fourth particle used for defining anisotropic polarizability. This may be set to -1, in which case aniso34 will be ignored. - **charge** (*double*) –   The charge on the Drude particle - **polarizability** (*double*) –   The isotropic polarizability - **aniso12** (*double*) –   The scale factor for the polarizability along the direction defined by particle1 and particle2 - **aniso34** (*double*) –   The scale factor for the polarizability along the direction defined by particle3 and particle4 |

    `setParticleParameters`(*self*, *index*, *particle*, *particle1*, *particle2*, *particle3*, *particle4*, *charge*, *polarizability*, *aniso12*, *aniso34*)¶
    :   Set the parameters for a Drude particle.

        |  |  |
        | --- | --- |
        | Parameters: | - **index** (*int*) – the index of the Drude particle for which to set parameters - **particle** (*int*) – the index within the System of the Drude particle - **particle1** (*int*) – the index within the System of the particle to which the Drude particle is attached - **particle2** (*int*) – the index within the System of the second particle used for defining anisotropic polarizability. This may be set to -1, in which case aniso12 will be ignored. - **particle3** (*int*) – the index within the System of the third particle used for defining anisotropic polarizability. This may be set to -1, in which case aniso34 will be ignored. - **particle4** (*int*) – the index within the System of the fourth particle used for defining anisotropic polarizability. This may be set to -1, in which case aniso34 will be ignored. - **charge** (*double*) – The charge on the Drude particle - **polarizability** (*double*) – The isotropic polarizability - **aniso12** (*double*) – The scale factor for the polarizability along the direction defined by particle1 and particle2 - **aniso34** (*double*) – The scale factor for the polarizability along the direction defined by particle3 and particle4 |

    `addScreenedPair`(*self*, *particle1*, *particle2*, *thole*) → int¶
    :   Add an interaction to the list of screened pairs.

        |  |  |
        | --- | --- |
        | Parameters: | - **particle1** (*int*) – the index within this Force of the first particle involved in the interaction - **particle2** (*int*) – the index within this Force of the second particle involved in the interaction - **thole** (*double*) – the Thole screening factor |
        | Returns: | the index of the screenedPair that was added |
        | Return type: | int |

    `getScreenedPairParameters`(*self*, *index*)¶
    :   Get the force field parameters for screened pair.

        |  |  |
        | --- | --- |
        | Parameters: | **index** (*int*) – the index of the pair for which to get parameters |
        | Returns: | - **particle1** (*int*) –   the index within this Force of the first particle involved in the interaction - **particle2** (*int*) –   the index within this Force of the second particle involved in the interaction - **thole** (*double*) –   the Thole screening factor |

    `setScreenedPairParameters`(*self*, *index*, *particle1*, *particle2*, *thole*)¶
    :   Set the force field parameters for screened pair.

        |  |  |
        | --- | --- |
        | Parameters: | - **index** (*int*) – the index of the pair for which to get parameters - **particle1** (*int*) – the index within this Force of the first particle involved in the interaction - **particle2** (*int*) – the index within this Force of the second particle involved in the interaction - **thole** (*double*) – the Thole screening factor |

    `updateParametersInContext`(*self*, *context*)¶
    :   Update the particle and screened pair parameters in a Context to match those stored in this Force object. This method provides an efficient method to update certain parameters in an existing Context without needing to reinitialize it. Simply call setParticleParameters() and setScreenedPairParameters() to modify this object’s parameters, then call updateParametersInContext() to copy them over to the Context.

        This method has several limitations. It can be used to modify the numeric parameters associated with a particle or screened pair (polarizability, thole, etc.), but not the identities of the particles they involve. It also cannot be used to add new particles or screenedPairs, only to change the parameters of existing ones.

    `usesPeriodicBoundaryConditions`(*self*) → bool¶
    :   Returns whether or not this force makes use of periodic boundary conditions.

        |  |  |
        | --- | --- |
        | Returns: | true if nonbondedMethod uses PBC and false otherwise |
        | Return type: | bool |

    `__delattr__`¶
    :   x.\_\_delattr\_\_(‘name’) <==> del x.name

    `__format__`()¶
    :   default object formatter

    `__getattribute__`¶
    :   x.\_\_getattribute\_\_(‘name’) <==> x.name

    `__hash__`¶

    `__reduce__`()¶
    :   helper for pickle

    `__reduce_ex__`()¶
    :   helper for pickle

    `__sizeof__`() → int¶
    :   size of object in memory, in bytes

    `__str__`¶

    `getForceGroup`(*self*) → int¶
    :   Get the force group this Force belongs to.

    `setForceGroup`(*self*, *group*)¶
    :   Set the force group this Force belongs to.

        |  |  |
        | --- | --- |
        | Parameters: | **group** (*int*) – the group index. Legal values are between 0 and 31 (inclusive). |

High performance molecular simulation on GPUs


### Navigation

- Application Layer
- Library Layer
  - Core Objects
  - Forces
  - Integrators
  - Extras

©2015, Stanford University and the Authors.
|
Powered by Sphinx 1.3.1
& Alabaster 0.7.7
|
Page source
